# Accurate Decoding of Short, Phase-Encoded SSVEPs

**DOI:** 10.3390/s18030794

**Published:** 2018-03-06

**Authors:** Ahmed Youssef Ali Amer, Benjamin Wittevrongel, Marc M. Van Hulle

**Affiliations:** 1 Electrical Engineering (ESAT) TC, Campus Group-T Leuven, Division Animal and Human Health Engineering, KU Leuven, 3000 Leuven, Belgium; ahmed.youssefaliamer@kuleuven.be; 2 Department of Neurosciences, Laboratory for Neuro- & Psychophysiology, KU Leuven, 3000 Leuven, Belgium; benjamin.wittevrongel@kuleuven.be

**Keywords:** BCI, EEG, SSVEP

## Abstract

Four novel EEG signal features for discriminating phase-coded steady-state visual evoked potentials (SSVEPs) are presented, and their performance in view of target selection in an SSVEP-based brain–computer interfacing (BCI) is assessed. The novel features are based on phase estimation and correlations between target responses. The targets are decoded from the feature scores using the least squares support vector machine (LS-SVM) classifier, and it is shown that some of the proposed features compete with state-of-the-art classifiers when using short (0.5 s) EEG recordings in a binary classification setting.

## 1. Introduction

The steady-state visual evoked potential (SSVEP) is an electrophysiological response to a periodically flickering visual stimulus that has been widely used to encode selectable targets in brain–computer interfacing (BCIs) [1,2,3] for assisted living- [4] and entertainment purposes [5,6,7,8,9]. When the subject directs his/her gaze to one of these targets, the electroencephalography (EEG) signal recorded from the subject’s occipital pole will resonate at the target’s frequency (and its harmonics). However, a simple frequency analysis based on the (fast) Fourier transform [10,11] typically requires long (i.e., 3 s or more [3,12]) signals to accurately discriminate targets flickering at nearby frequencies. Recent research has focused on the accurate discrimination of targets based on short signals [13,14,15,16,17,18].

The number of frequency-encoded targets that can be displayed is limited by the display’s refresh rate, the harmonics of the flickering targets, which tend to quickly crowd the frequency spectrum, and the subject’s optimal frequency range. This has called for another variable to encode targets. Several authors have suggested using phase-encoding: targets adopt the same frequency but are encoded with different phases [19,20,21,22,23], or each target is encoded with a unique combination of frequency and phase [13,14,16,24]. Although this method is theoretically sound, in practice there is no fixed relation between the phase lag of the EEG recordings and the stimulation phase. The measured phase is inter-subject, inter-electrode, and inter-session-dependent [25]. Phase estimation can be based on frequency domain analysis [24], but this requires long stimulation times to be sufficiently accurate. Because of this drawback, other methods have been developed, such as an extension of canonical correlation analysis (CCA) [14] and spatiotemporal beamforming [18], that were shown to discriminate four joint frequency-phase encoded targets with a maximal median accuracy between 90% and 92% using 0.5-s stimulation times [16].

In this study, several novel features for phase-encoded SSVEP target identification are proposed in the context of short stimulation times (0.5-s) in order to speed up BCI communication.

## 2. Methods

### 2.1. Experimental Data

The EEG data used in this study was obtained from a previous study of our group [16]. In the original study, 21 subjects (14 female, average age of 22.7 years, ranging from 19 to 26 years) participated in an SSVEP experiment that used four targets, each one encoded with a unique combination of frequency (12 or 15 Hz) and phase (0 and π radians). During the experiment, subjects were instructed to direct their gaze to a cued target and to maintain their focus during the subsequent 5-s stimulation when all targets were simultaneously flickering at their assigned frequency-phase combinations that were rendered by sinusoidally modulating their luminosities [23]. Each target was cued 15 times. EEG was recorded continuously (sampling speed 2048 Hz, common mode sense (CMS) referencing) using 32 active Ag/AgCl electrodes (BioSemi Active Two) that were placed according to the extended international 10–20 system. Additionally, two electrodes were placed on the mastoids for offline referencing, and four electrodes around the eyes for offline electrooculogram (EOG) correction using the RAAA-procedure [26].

### 2.2. Signal Preprocessing

The raw signals were offline re-referenced to the average mastoid signal, and the EOG correction [26] procedure applied. The eye-artifact corrected signals were then band-pass filtered between 5 and 20 Hz using a 4th order Butterworth filter, cut into five-second epochs, time-locked to the stimulation onset, downsampled to 512 Hz, and labeled with the corresponding target. For each subject, 60 five-second epochs (2560 sample-points per epoch) were extracted, from which we only retained the first 0.5 s (256 sample-points) for further analysis. Furthermore, the analysis was limited to the three occipital electrodes (i.e., Oz, O1, and O2) and the trials divided into two datasets based on their stimulation frequencies. Each dataset, thus, contained 30 phase-encoded trials, but for different frequencies (12 and 15 Hz).

### 2.3. Feature Extraction

The following four feature extraction methods, new to SSVEP BCI, were considered to distinguish the EEG signals of the two flickering targets with opposite phase.

Method I: Estimated phase based on the maximum likelihood condition: The intuition behind this feature is to treat the SSVEP BCI set-up as a telecommunication system that relies on phase shift keying (PSK), a coding technique based on modulating the carrier signal’s phase. The phase for epoch $r_{e}\in R^{1\times n}$, where *n* (=256) represents the samples in time, was estimated by maximizing the likelihood of the $r_{e}$, given the flickering stimulus $\sin(2\pi f_{c}t)$, as follows [27]:
  (1) $${\hat{\varphi }}_{ML}=-\tan^{-1}\frac{\int_{T_{0}}r\left(t\right)\sin\left(2\pi f_{c}t\right)dt}{\int_{T_{0}}r\left(t\right)\cos\left(2\pi f_{c}t\right)dt}$$
  where ${\hat{\varphi }}_{ML}$ represents the maximum likelihood, $f_{c}$ is the stimulation frequency (12 or 15 Hz), and $T_{0}$ is the time period of the epochs (i.e., 0.5-s).
Method II: Correlation with 0/π phase templates: From the training set, a template for each target i∈[1..2] was obtained by applying singular value decomposition (SVD) to $E_{i}\in R^{k\times n}$, the matrix containing all *k* training epochs having target *i* as label. The template for target *i* was given by the component corresponding to the largest singular value.
  For each epoch $r_{e}$, two features were then given by the (Pearson) correlation coefficients between $r_{e}$ and both templates.
Method III: Phases of the 0/π templates: Similar to the previous feature, first, the training data was used to obtain a template for each target using the same SVD procedure as before. However, instead of calculating the correlation, this time the features were given by estimating the phase (using the ML method, Equation (1)) between an epoch $r_{e}$ and the two templates.
Method IV: Correlation of one-period segments: In this method, a correlation coefficient with a reference signal was calculated, but unlike before, the reference signal was not a full epoch. The procedure is explained next.
  First, following the time-domain analysis of SSVEP [16,18,28,29], each training epoch $r_{e}$ was cut into consecutive, non-overlapping segments with length one period of the stimulation frequency after which the segments were averaged to obtain ${\hat{s}}_{e}\in R^{1\times x}$, where *x* is the length of one period of the stimulation frequency. In our case, the segment length (*x*) was equal to 1/12 or 1/15 s and, for each epoch, 6 or 7 segments were extracted and averaged, depending on the dataset under consideration.
  Next, the Pearson correlation coefficient matrix $R\in R^{e\times e}$ of the averaged segments was calculated as follows:
  (2) $$R\left[i,j\right]=pearson\left({\hat{s}}_{i},{\hat{s}}_{j}\right)$$
  where *e* is the number of (training) trials. Using this matrix, several segments were selected that will serve as a reference for the correlation with novel data. The following references have been selected:
  - one segment corresponding to the column from R that maximizes the correlation with the class labels;
  - two segments, one for each target, that we selected from the column with the most centered mean of the correlation coefficient values, which indicates the epoch that can be considered a reference for the same target epochs;
  - two segments based on data statistics (i.e., standard deviation): the two columns with maximum and minimum standard deviations.
  For each epoch $r_{e}$, five features were extracted, given by the (Pearson) correlation of ${\hat{s}}_{e}$ with all references.

### 2.4. Feature Combination and Signal Alignment

The feature extraction methods mentioned above were applied to the three occipital signals individually. As for Methods I, II, and III, the three occipital signals were additionally aligned using the singular value decomposition (SVD) [30,31] for each individual trial. This aligned signal was treated as an additional channel. Hereto the three-channel epoch is represented in the rows of matrix $X\in R^{3\times n}$, and by applying [U,S,V]=SVD(X), with U the left singular vectors, S the singular values diagonal matrix, and $V^{⊺}$ the right singular vectors. The singular vector corresponding to the largest singular value was extracted, and the signal was then recomposed to obtain the aligned epoch. As far as we are aware, this SVD alignment method has never been used in EEG studies, albeit it has recently also been described in the context of functional magnetic resonance imaging (fMRI) [32].

For each epoch, the features of all considered signals are grouped into a feature set (FS). For example, FS-I contains the features of all four signals using the first feature extraction method (i.e., FS-I contains four phase estimates). The dimensions for each feature set (i.e., the number of extracted features per epoch) are given in Table 1.

In what follows, the performances of all possible feature set combinations (exhaustive search) are explored.

### 2.5. Classification & Performance Evaluation

In order to assess the phase-decoding performance of the proposed procedures, the least squares support vector machine (LS-SVM) [33] was used with a stratified 5-fold cross-validation. The parameters of the LS-SVM model were tuned using the simplex optimization function and a stratified 10-fold cross validation on the training set. We repeated this procedure for both the 12 and 15 Hz datasets and compared our results with two state-of-the-art classifiers: the spatiotemporal beamformer (stBF) [16,18,34,35] and extended CCA (eCCA) [14] methods. Both stBF and eCCA were given the three occipital channels as input, without the SVD-aligned signal. Because of the inter-subject variation in phase response [16], the analysis was run for each subject individually.

In recent years, many studies have investigated the simultaneous frequency-phase encoding of the selectable targets [13,14,16,24]. Hence, the predictive properties of our features for the decoding of all four frequency-and-phase encoded targets was additionally investigated. To that end, a stratified 5-fold cross-validation was run on the full dataset, and from the training data, two LS-SVM classifiers were trained, each one tailored to the decoding of the phase-encoded target of one of the frequencies (12 and 15 Hz), as before. The prediction of a testing trial was then done by extracting features twice, each time assuming one of the two frequencies, and making a prediction using the corresponding classifier. In addition to the prediction, the LS-SVM also returns the posterior probability that can be viewed as the confidence of the classifier towards the prediction. If the extracted features only result in meaningful values when the assumed frequency is equal to the actual frequency, simply running the trial through both classifiers should result in a confident prediction of one classifier, while the confidence of the other is considerably less. The most confident prediction was then taken as the winner.

Finally, also the time-complexity of the proposed feature extraction methods was assessed to verify its feasibility for deployment in a real-time setting. The time needed to finish training the classifiers was measured on a quad-core Intel i7 (Santa Clara, CA, USA) machine.

### 2.6. Statistics

A paired Wilcoxon signed-rank test was applied to compare the performance of the best proposed method and the two state-of-the-art classifiers. Bonferroni correction was applied to account for multiple comparisons, as three conditions were compared. *P*-values below the (corrected) threshold of 0.016 (=0.05/3) were considered significant.

## 3. Results

**Figure 1:**
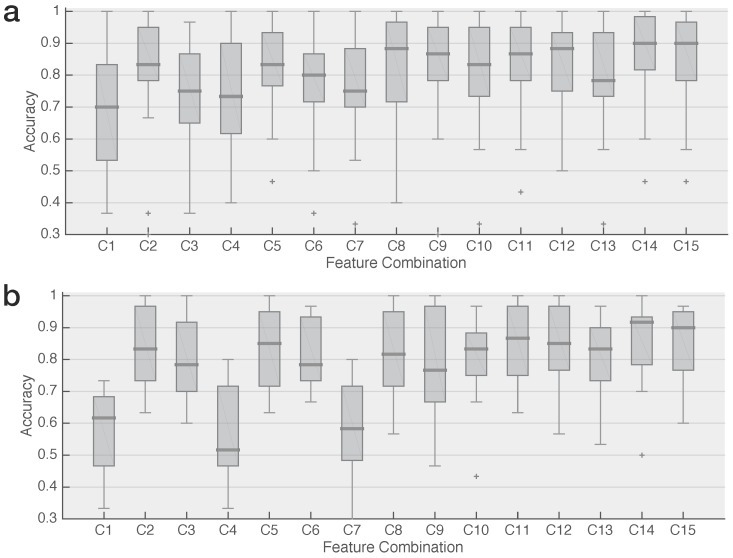
Accuracy of target identification using all possible combinations of four features for a recording period of 0.5 s for (**a**) two 12 Hz phase-encoded targets and (**b**) two 15 Hz phase-encoded targets. The accuracies of all feature combinations are summarized in boxplots: the thick horizontal line indicates the median value, the box stretches from the 1st to the 3rd quartile, the lines extending from the box indicate the minimum and maximum value within 1.5 times the interquartile range from the 1st and 3rd quartile, respectively, and the plus-signs represent outliers.

The classification accuracies of the LS-SVM for all possible features combinations for both phase-encoded datasets are visualized in Figure 1. Given the four features, there are 15 possible ways to combine them, numbered from 1 to 15. The numbering can be found in Table 2.

For the 12 Hz dataset, all feature combinations reach a (median) accuracy of 70%, which is often deemed necessary to establish reliable communication [18,36,37,38,39,40,41], while, for the 15 Hz dataset, C1, C4, and C7 are not able to reach this threshold (Figure 1). The combinations with the highest accuracies are C14 and C15, which obtain a median performance of 90% or more for both datasets. From these two, C14 seems to be the better combination as it reaches a slightly higher accuracy for the 15 Hz dataset and has a smaller inter-subject variability for both datasets.

**Figure 2:**
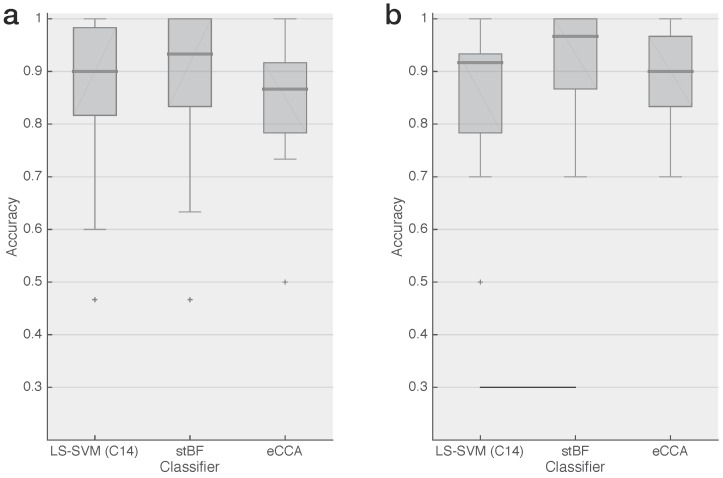
Classification accuracy of the best proposed feature set (C14) with LS-SVM compared with spatiotemporal beamforming and extended CCA for an epoch length of 0.5-s for (**a**) two 12 Hz phase-encoded targets and (**b**) two 15 Hz phase-encoded targets. The accuracies are summarized in boxplots using the same convention as in Figure 1. The black horizontal lines at the bottom of the figure indicate significant differences based on the paired Wilcoxon signed-rank test with Bonferroni correction.

Figure 2 shows the classification accuray of the best feature combination (C14) with LS-SVM and with two state-of-the-art classifiers for both datasets. There is no significant difference in target identification accuracy between the proposed method and eCCA for both datasets, and only for the dataset with the 15 Hz encoded targets is there a significant difference between the proposed method and stBF.

From a time complexity perspective, all possible feature set combinations are similar, with an average elapsed time of 0.010 s. In comparison, the times needed to train the eCCA and stBF classifier are 0.002 and 0.042 s, respectively.

**Figure 3:**
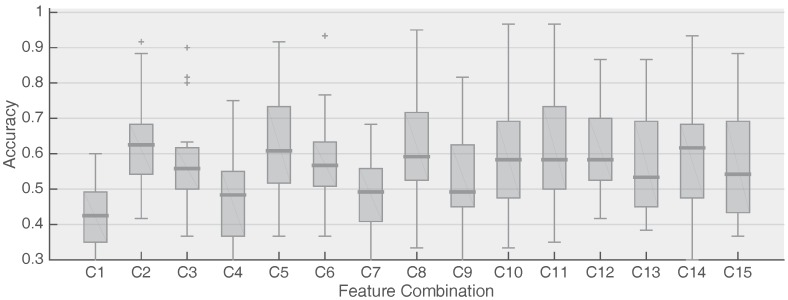
Classification accuracy of the proposed features in the case of frequency-and-phase-encoded targets. Two classifiers were trained, each one tailored to detect phase-encoded targets of a given frequency (12 and 15 Hz in our case). For each trial, features were extracted twice (each time assuming the presence of one of the frequencies) and fed into the corresponding classifier. The prediction of the classifier with the highest confidence (based on the posterior probability) was taken as the winning target.

Figure 3 shows the accuracy of the decoding of all four frequency-and-phase encoded targets.

None of the feature combinations provide sufficient accuracy to establish a reliable communication channel using frequency-and-phase-encoded targets, indicating that a dedicated frequency feature or a prior frequency-selection step will be necessary to adopt the proposed features in the context of simultaneous frequency-and-phase decoding.

## 4. Conclusions

In this study, several novel features for discriminating phase-encoded SSVEP targets were proposed. All combinations of the proposed features were exhaustively investigated, and it was shown that the best feature combination, in combination with an LS-SVM, performs comparably to two state-of-the-art SSVEP classifiers, and that the time complexity of the feature selection is sufficiently low to allow for real-time decoding.

While the proposed features were able to accurately decode two phase-encoded targets for two base frequencies (12 and 15 Hz), the results show that they are not informative enough for accurate frequency-and-phase decoding. We therefore propose the development of frequency-specific features or the adoption of a prior frequency-selection procedure.

## Figures and Tables

**Table 1:** Feature vector dimensions (i.e., number of extracted features) per epoch for each feature set.

Feature Set	FS-I	FS-II	FS-III	FS-IV
Dimensions per epoch	4	8	8	15

**Table 2:** Exhaustive list of all feature combination with their combination reference.

Combination	Feature Set (FS)			
	I	II	III	IV
C1	x
C2		x
C3			x
C4				x
C5	x	x
C6	x		x
C7	x			x
C8		x	x
C9		x		x
C10			x	x
C11	x	x	x	
C12	x	x		x
C13	x		x	x
C14		x	x	x
C15	x	x	x	x
